# Elements of Physics for Medical Students

**Published:** 1907-12

**Authors:** 


**Elements of Physics for Medical Students.** By FREDERIC J. M. PAGE, B.Sc., F.I.C. Pp. xvi., 288. London: Cassell and Company, Limited. 1907.

The author describes this volume as a text-book which, in conjunction with the *Manual of Chemistry* by Luff and Page, recently noticed in our columns, serves to cover all the chemistry and physics—both theoretical and practical—required by the Conjoint Board and the Society of Apothecaries. In reality it is a well-illustrated syllabus, and excellently adapted to the needs of the teacher, and just sufficient to refresh the mind of the apt and well-taught student. Without simultaneous expansion, by precept and practical demonstration, it would be unintelligible to the beginner in the science of physics. Of this no doubt the author is well aware, but the claim to be a text-book is scarcely justified. On reading the book, one is impressed with the nakedness of our school education of the present day, which sends boys into the study of medicine ignorant of the simple principles of physics which this syllabus includes, and makes it necessary to spare some of the all too short five years' curriculum in teaching such rudiments of knowledge (we forbear to designate it as a special branch of learning) as can readily be grasped by any boy of thirteen not deficient.

It is incredible that a youth can register as a student of medicine ignorant of the facts contained in this superficial survey, which after all includes nothing but a few of those generalisations based on observation which pass under the name of laws, *e.g.* Boyle's Law, Avogadro's, Gay Lussac's, Ohm's, and the like. Still, we must take our author's word for it, this kindergarten standard is the one required by our chief examining boards. From this point of view, it must be admitted that most of the statements in the book are accurate, for to have misquoted the "laws of reflection" would be scarcely possible, and the whole work is little more than a series of such quotations. In many places brevity has proved the handmaiden of obscurity, above all in the chapter where it was least excusable, that on "Light." The explanation of the *diopter* is an example of a sentence so compressed as to defy unravelling ; one must merely begin afresh and make a new statement in order to be intelligible to the unlearned.

There is also a poor little sentence crying in the wilderness, saying, "Concave lenses would be—2 D, etc." Disowned by the context on either side, it is difficult to fill up the hiatus of that "&c." Myopia is undoubtedly described misleadingly, being attributed entirely to defects of the lens when no theory of causation need have been introduced.

Acoustics merely left us wondering—thirteen and a half pages wasted ! Here even the supposition that the volume might prove useful as a syllabus in the hands of the teacher failed; but we began to appreciate why the art of auscultation and percussion is so mysterious to the average student, and that to try to build up a superstructure of knowledge on a foundation is a task foredoomed to failure.

As a mere outline of Mr. Page's course of instruction we say no ill of it, but as a student's hand-book it is not worthy of consideration.

